# A Comparison of the Effects of Acupressure and Music on Venipuncture Pain Intensity in Children: A Randomized Controlled Clinical Trial

**DOI:** 10.1155/2024/2504732

**Published:** 2024-01-18

**Authors:** Faezeh Daihimfar, Hassan Babamohamadi, Raheb Ghorbani

**Affiliations:** ^1^Student Research Committee, Semnan University of Medical Sciences, Semnan 3513138111, Iran; ^2^Nursing Care Research Center, Semnan University of Medical Sciences, Semnan 3513138111, Iran; ^3^Department of Nursing, School of Nursing and Midwifery, Semnan University of Medical Sciences, Semnan 3513138111, Iran; ^4^Social Determinants of Health Research Center, Semnan University of Medical Sciences, Semnan, Iran; ^5^Department of Social Medicine, School of Medicine, Semnan University of Medical Sciences, Semnan 3513138111, Iran

## Abstract

Pain from injections is common in children of all ages, and more than 90% of hospitalized children experience invasive and painful procedures such as venipuncture. In light of the complications associated with pain relief medications, nonpharmacological and complementary medicine approaches have gained attention. This study aims to compare the effects of acupressure and music on venipuncture pain intensity in children. This randomized controlled clinical trial involved 180 children aged 3–6 years who sought treatment at the Children's Medical Center Hospital Emergency Department at Tehran University of Medical Sciences, Iran. The children were randomly assigned to one of three groups: acupressure, music, or control. The interventions were given within 5 minutes, starting 3 minutes before the venipuncture and continuing until completion. The interventions included playing music through headphones and applying acupressure to the Hugo point. Venipuncture was carried out under identical conditions using an Angiocath 24G needle. Pain intensity was assessed using the Oucher scale. Data were analyzed using SPSS 24, employing the Kruskal–Wallis, chi-square, and Bonferroni pairwise comparison tests, with a significance level of 0.05. The mean pain intensity was 3.32 ± 1.44 in the music group, 4.82 ± 1.51 in the acupressure group, and 8.32 ± 1.10 in the control group. Pain intensity significantly differed among the three groups (*p* < 0.001). Specifically, pain intensity was lower in the music group compared to both the acupressure (*p* < 0.001) and control (*p* < 0.001) groups. Furthermore, pain intensity was lower in the acupressure group than in the control group (*p* < 0.001). Based on the results, music and acupressure methods effectively reduce pain intensity during venipuncture in children. Considering that music demonstrated a more pronounced effect in alleviating venipuncture pain than acupressure, the recommendation is to utilize music as a method of pain management during venipuncture in children. Iranian Registry of Clinical Trials, Trial No. IRCT20120109008665N15, was registered on 6 December 2021.

## 1. Introduction

The word pain originates from the Latin word “*poen*” or the Greek word “*INEPO*,” meaning penalty and punishment [1]. It is a common cause of suffering experienced by 50–80% of hospitalized patients. This complication causes discomfort, distress, and disability more than any other diseases [2]. Pain is a symptom and should be treated independently, not just as an inevitable consequence of a disease [3]. It is an essential and common health problem, the fifth vital sign, and the most critical symptom in hospitalized children [4].

Based on feedback from children admitted to the pediatric departments, venipuncture is reported as the primary source of pain in the hospital following surgical procedures. Unfortunately, venipuncture is a common clinical practice for children [5]. More than 90% of hospitalized children experience painful, invasive procedures (e.g., venipuncture) [6]. Hospitalized children consider the pain of the venipuncture needle to be horrifying and a significant source of pain in the hospital [7]. On the other hand, unnecessary pain causes children to lose trust in the nurse, while trust is a prerequisite for communication and acceptance of therapeutic measures [8].

Children are usually afraid of syringes and deny their pain due to their fear [9]. Children's pain caused by medical procedures leads to short-term suffering. However, recent data indicate that such pain has long-term and stable adverse effects in such a way that it causes fear, stress, and anxiety before venipuncture and avoiding medical care in adulthood [10]. Injection pain is joint in all childhood ages. According to a study of 242 children hospitalized, 49% of them stated that the discomfort due to needles and injections was worse than other procedures during their hospitalization [11].

Many nurses ignore acute pain evaluation, management, and treatment [12]. In contrast, pain evaluation is a crucial part of the nursing process, and nurses should consider the child's comfort as a basic need and use different methods to decrease and relieve their pain to the minimum possible level [13]. Pharmaceutical and nonpharmacological methods are recommended to relieve children's pain [14], but the effectiveness of some methods is still questioned [15].

Aromatherapy with lavender, massage therapy, rhythmic breathing, relaxation techniques, piroxicam gel, ethyl chloride spray, lidocaine spray, Valsalva maneuver, acupressure, and thought distraction are some pain relief methods reported in the literature [16–21]. There are different methods to decrease venipuncture pain, one of which is Hugo point acupressure, which has been investigated in many studies [22–25]. Acupressure refers to the stimulation of acupuncture points using fingers [26].

One of the acupressure points is the large intestine meridian (LI4). It is the most crucial point for pain relief and is on the back of the hand in the middle of the first and second metacarpals' bisectors [27, 28]. Acupressure at this point improves lymph and blood flow, decreases muscle stiffness, and prevents interstitial fluid stagnation, thereby accelerating the purification of blood and lymph, ultimately increasing the blood oxygen level and providing more oxygen and nutrients to cells [29]. This point is located where the energy flow is closer to the skin surface and can be easily stimulated by pressure, a needle, or extreme cold [30].

Several studies have investigated the effect of acupressure on pain due to venipuncture, vaccination, or fistula cannulation in dialysis patients. For example, Baloochi et al. studied the effect of SP6 and ST36 acupressure on pain intensity due to fistula cannulation in hemodialysis patients. They concluded that effectively massaging these points was a safe and simple way to reduce patient pain [31]. Khosravan et al. investigated the effect of Hugo point massage with and without ice on the vaccination pain intensity in infants. The researchers concluded that Hugo point massage with and without ice effectively relieved pain [30]. In a study, Lang et al. concluded that acupressure was effective in reducing pain and anxiety in patients with radial fracture [32].

Distraction is a noninvasive way of controlling pain during venipuncture [9]. Music therapy is a health profession based on evidence and art that uses music as a therapeutic method to fulfill physical, emotional, cognitive, and social needs. Music is effective for all ages, from childhood to adulthood, and in all healthcare issues, including chronic diseases and social fields [33]. It affects pain in two ways: attracting the patient's listening attention, engaging the mind in music, and distracting from pain [34, 35]. The second way, which is more fundamental, is the effect of music on the release of brain morphine, cortisol, and stress hormones [36].

Many studies have investigated the effect of music on reducing pain intensity and anxiety and creating relaxation [37–40]. A study indicated that the use of thought distraction methods (solving riddles and music) decreased the pain intensity caused by opening the intravenous line (IV line) in children [41]. A study conducted by Aydin et al. reported that there were no significant differences in pain levels among the three intervention groups across three assessment modes: self-report, parents' report, and control (*p*=0.15, *p*=0.232, and *p*=0.72). Furthermore, they reported a high level of pain in the control group [42]. In a study investigating the effect of thought distraction on pain, fear, and discomfort during intravenous catheter insertion in children and adolescents with cancer, Windich et al. concluded that pain was reduced in the intervention group. However, there was no significant difference among the groups [43].

Despite various methods in this field, ongoing research is being conducted on the most suitable pain relief methods that are effective and affordable. Given that pain relief in sick children is a part of their rights, nurses must take every suitable measure to relieve their pain [44]. Ignoring the effects of painful procedures in children leads to short and long-term irrecoverable complications [22]. Researchers have not yet reached a consensus on the preferable method to reduce or relieve children's pain, and the results of studies are sometimes contradictory. Hence, further investigation is required to identify effective methods. Consequently, the current study seeks to compare the efficacy of acupressure at the Hugo point with the soothing influence of music in alleviating venipuncture-induced pain in pediatric patients.

## 2. Materials and Methods

### 2.1. Study Design and Participants

**Figure 1:**
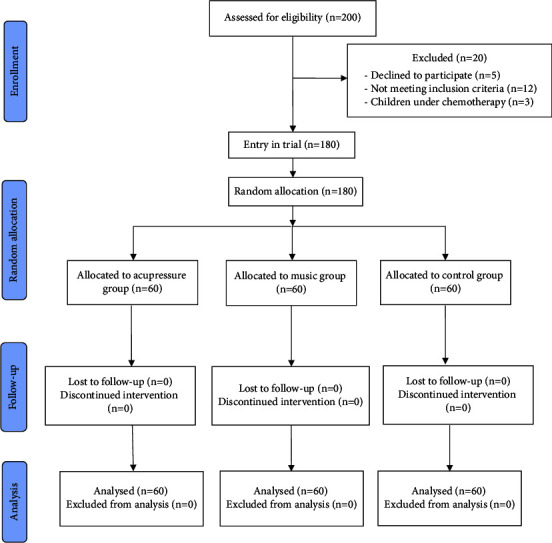
CONSORT flowchart of the study.

The current study was a parallel randomized clinical trial conducted on children aged 3 to 6 years who required venipuncture at the Children's Medical Center Hospital, affiliated with Tehran University of Medical Sciences in Iran, from November 2021 to the beginning of February 2022. A pilot study was carried out to determine the sample size. In this regard, 10 samples were selected from each group: acupressure, music therapy, and control. The mean pain intensity, represented as mean ± standard deviation, was 3.1 ± 32.44 in the children of the music therapy group, 4.82 ± 1.51 in the acupressure group, and 8.32 ± 1.10 in the control group. The sample size was estimated at 60 per group, considering a 95% confidence interval, 80% test power, and the equation *n*=(*z*_1−*α*/2_+*z*_1−*β*_)^2^ × (*δ*_1_^2^+*δ*_2_^2^)*/*(*μ*_*1*_ *−* *μ*_*2*_)^*2*^. The effect size was determined to be 1.05 using G*∗*power software. A total of 200 individuals were initially included in the study, with 20 individuals ultimately excluded for various reasons, leaving 180 patients for analysis (60 per group). The convenience purposive sampling method was employed for participant selection. Children meeting the inclusion criteria were randomly assigned to one of three groups: control, acupressure, and music therapy. A total of 180 sealed envelopes, divided equally between 90 girls and boys, containing the letters A (acupressure), M (music), and C (control), were used to allocate children to the respective groups. The children themselves selected the envelopes. Age and gender were matched among the sampled children. Blinding was not feasible due to the ease of identifying patients receiving acupressure or music therapy; thus, this study employed an open-label approach (Figure 1).

The inclusion criteria were as follows: children aged 3 to 6 years requiring venipuncture, conscious state, parental consent for participation, suitability for engaging in massage and listening to music, parents not belonging to the medical and health personnel group, relative physical and mental health, and not undergoing chemotherapy. Exclusion criteria encompassed the use of sedatives, hypnotics, and painkillers, unsuccessful initial cannulation requiring repetition, seizures or life-threatening conditions during the study, and critically ill patients for whom assessing pain intensity without the confounding effect of acute illness was not feasible based on their disease severity.

### 2.2. Ethical Considerations

Ethical considerations were given due attention in the conduct of this study. Approval for this study was granted by the Ethics Committee of Semnan University of Medical Sciences (IR.SEMUMS.REC.1400.222), and it was duly registered in the Iranian Registry of Clinical Trials (IRCT20120109008665N15). Data collection commenced following the requisite permissions from the Children's Medical Center Hospital authorities.

At the outset of the study, after visiting the center and conducting an introductory session with the research subjects, the researchers elucidated the research objectives and methodologies. They reassured the participants regarding the confidentiality of their information and emphasized the voluntary nature of their participation in the study. Any questions raised by the participants were duly addressed. Following this, written informed consent to participate in the study was obtained from the parents of the children involved.

### 2.3. Interventions

In the Hugo point acupressure group, children received acupressure at the Hugo point for 5 minutes, starting 3 minutes before the commencement of venipuncture and continuing until the conclusion of the procedure. This acupressure technique was administered by a trained nurse, who used the tips of either the index fingers or thumb to apply a rotating pressure to the Hugo point. Consequently, the Hugo point on the child's noncannulated hand was subjected to moderate anticlockwise rotational pressure. A 30-second break was provided for every 1 minute of pressure application, and this procedure was sustained for the entire 5-minute duration until the venipuncture was completed.

Children in the music group were exposed to a cheerful instrumental children's song via headphones, by Iranian cultural preferences, for 5 minutes, beginning 3 minutes before the conclusion of the venipuncture procedure.

No special intervention was performed on the control group, and they received only routine care measures.

A nurse performed venipuncture in a dedicated venipuncture room under consistent environmental conditions that were identical for all children. To ensure uniformity across all three groups, the presence of parents was not permitted during the procedure. Additionally, the nurse was trained to maintain uniform behavior and speech when interacting with all the participants.

For the venipuncture procedure, a yellow Angiocath 24G from the Vitroflon brand was utilized in the brachial region of the children's hands. The participant was excluded from the study if venipuncture was unsuccessful in the initial attempt.

A single evaluator, distinct from the individual responsible for Angiocath insertion, assessed pain intensity for all patients. This evaluation was performed at the moment when the Angiocath was inserted into the child's body.

### 2.4. Data Collection

A two-part questionnaire was employed for data collection. The initial segment encompassed demographic information, including underlying medical conditions, the rationale for referral, age, gender, and previous history of intravenous injections. To acquire demographic data, the researcher consistently frequented a specific healthcare center in collaboration with a seasoned nurse. After elucidating the research's objectives, establishing trust, securing consent from the children, and obtaining written parental consent, the researcher recorded the demographic details and incorporated the child into the study.

The second part comprised the Oucher Child Pain Assessment Scale. Immediately after the venipuncture procedure, the Oucher scale was administered to all children in the three groups through self-reporting. This scale is a numerical-visual instrument tailored for children aged 3–12 years and is structured with two columns placed side by side, featuring numbers ranging from zero to one hundred alongside six pictures. On this scale, zero indicates the absence of pain, while 1–29 denotes mild pain, 30–69 represents moderate pain, 70–99 signifies severe pain, and 100 corresponds to the utmost pain (notably, a cutoff point of 30–69, signifying moderate pain, was utilized in this research). The numerical section was used for children with numerical skills, while the visual component was employed for those without such skills. In the numerical range of 0–100, the numbers specified by the children indicated their pain scores.

**Figure 2:**
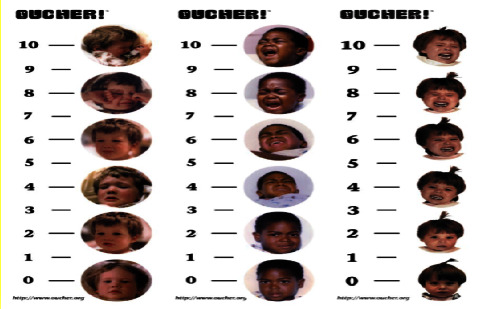
Oucher scale.

When employing a visual scale, the images selected by the children were converted into scores using even numbers ranging from 0 to 100, with the first image corresponding to 0, the second image to 20, the third image to 40, the fourth image to 60, the fifth image to 80, and the sixth image to 100 (Figure 2).

Due to the resemblance of the Spanish version's images to those of Iranian children, the Spanish version was adopted in the current study [45]. The validity and reliability of the Oucher scale have been substantiated in numerous studies [46–48]. Beyer et al. reported the tool's reliability as 0.947 for the numerical scale and 0.912 for the visual scale, with a significance level of *p* < 0.001 [49]. Sharifian et al. attested to the instrument's reliability, yielding a coefficient of 0.89 using Kendall's coefficient, thus indicating a high level of reliability [50]. In this study, the instrument's reliability was assessed via the inter-rater method, resulting in an 85% concordance coefficient, indicative of a commendable instrument reliability.

### 2.5. Statistical Analysis

The data analysis was conducted utilizing the per-protocol approach within SPSS 24, maintaining a significance level of 0.05. Frequency tables were constructed to describe, categorize, and compare the research data. Additionally, the Kolmogorov–Smirnov test was employed as an initial step to assess the normality of the data distribution and conduct data analysis.

The chi-square test was employed to juxtapose the absolute and relative frequencies of the research participants concerning gender, medical history, reasons for hospital visits, and prior injection history. Furthermore, for age-related comparisons, the Kruskal–Wallis test was applied. This test was also utilized to assess the mean pain intensity among the research participants.

To delve further into the mean pain intensity during venipuncture across the three groups, the Bonferroni pairwise comparison post hoc test was employed.

## 3. Results

### 3.1. Participants' Characteristics

The present study examined 180 children undergoing venipuncture. The mean age of the children was 4.28 ± 1.23 years in the music therapy group, 4.62 ± 1.11 years in the acupressure group, and 4.50 ± 1.12 years in the control group. Most children in the music therapy and acupressure groups (51.7%) and 50% of the children in the control group were females.  Table 1 provides the demographic information of the research subjects. The three groups did not exhibit significant differences in demographic variables (*p* > 0.05).

### 3.2. Pain Intensity

In the context of the absolute and relative frequency distribution of research units concerning pain intensity during venipuncture, the findings reveal that most units experienced moderate pain in the music therapy group (60%) and the acupressure group (78.3%). Conversely, the control group exhibited very severe pain in 15% of cases and severe pain in 80% of cases. Notably, none of the children in the music therapy and acupressure groups reported severe pain. Regarding pain intensity during venipuncture, the lowest mean pain intensity was observed in the music therapy group (3.32 ± 1.44), while the highest was documented in the control group (8.1 ± 32.10). The acupressure group reported a mean pain intensity of 4.82 ± 1.51. Statistical analysis using the Kruskal–Wallis test demonstrated a significant difference in pain intensity among the three groups during venipuncture (*p* < 0.001). Specifically, the pain intensity in the music therapy group was significantly lower than that in the acupressure group (*p* < 0.001) and the control group (*p* < 0.001). Furthermore, the acupressure group experienced less pain intensity compared to the control group (*p* < 0.001) (Table 2).

Regarding the nonhomogeneous nature of the three groups concerning their history of previous injections, a separate pain analysis was conducted for the subgroups with and without previous injections.

- The pain intensity significantly differed among the three groups in the subgroup with a history of previous injections(*p* < 0.001). Notably, the pain intensity was lower in the music therapy group compared to both the acupressure group (*p*=0.027) and the control group (*p* < 0.001). Additionally, the acupressure group experienced lower pain intensity than the control group (*p* < 0.001).
- A similar pattern emerged in the subgroup with no history of previous injections, with a significant difference in pain intensity among the three groups (*p* < 0.001). Once again, the music therapy group reported lower pain intensity compared to both the acupressure group (*p*=0.001) and the control group (*p* < 0.001). Furthermore, the acupressure group exhibited lower pain intensity than the control group (*p* < 0.001) (Table 3).

## 4. Discussion

The results of the present study, aimed at comparing the effects of acupressure and music therapy on venipuncture pain in children aged 3–6, indicate that music therapy provides more effective pain relief than both the acupressure and control groups. Additionally, pain intensity was lower in the acupressure group compared to the control group. To the researcher's knowledge, no clinical trial has previously compared the effectiveness of acupressure and music therapy on venipuncture pain intensity.

In the current study, the mean pain intensity following venipuncture was significantly lower in the music therapy group and then in the acupressure group, respectively, compared to the control group. The results also revealed a significant difference in pain intensity among the three groups. Pairwise comparisons of mean pain intensity also indicated significant differences among the three groups, both with and without a history of previous injection. Numerous studies have investigated the effect of music in reducing pain intensity and anxiety and inducing relaxation [37, 38, 51, 52]. Consistent with these findings, Esmaeili et al. conducted a study on 36 samples of thalassemia boys and girls aged 6 to 12 years. Their results indicated that venipuncture pain was significantly lower during breathing exercises and music therapy than in the control group. In this regard, music significantly contributed to pain reduction (*p*=0.0001) [53]. In a study, Evans et al. found that music therapy was an effective complementary medicine method for all age groups and could be applied in cases where children experienced acute and chronic pain, such as venipuncture, circumcision, burns, and surgeries.

Furthermore, active music therapy involving the child's participation proved more effective than music therapy played with headphones or live music [54]. Shahabi et al. conducted a study on 46 thalassemia children of school age. Their findings concluded that venipuncture pain was lower in the music therapy and EMLA cream groups than in the control group (*p* < 0.001). However, the pain experienced by children while using music was not significantly different from the use of EMLA cream [55].

In contrast to the above findings, Pourmovahed et al.'s study reported that the mean pain intensity score was lower in the EMLA cream group (1.36) compared to the music therapy group (3.5) and the control group (3.56). Furthermore, the pain intensity was lower in the music group compared to the control group, but the difference was not statistically significant [56]. This difference in outcomes may be attributed to the age group of the children involved.

Moreover, it is possible to compare this method with EMLA cream, which, due to cutaneous absorption, can induce analgesia in the superficial skin layers up to a depth of 5 mm [57, 58]. The results of a meta-analysis conducted by Fetzer et al., which included 20 studies and recommended using EMLA cream for venipuncture due to its potential for pain relief, support this distinction [59].

The utilization of music in patient care can be categorized into different approaches, ranging from patient-initiated music listening to music medicine (where medical personnel provide prerecorded music for symptom management) and music therapy (which involves the psychotherapeutic use of music). Various authors have emphasized the need to distinguish between these practices when studying the effectiveness of music interventions [60, 61]. Music medicine (MM) does not involve a structured therapeutic process, whereas music therapy (MT) necessitates establishing a therapeutic relationship between the client and a trained music therapist. This involves personalized music experiences such as listening to live or prerecorded music, playing musical instruments, improvising, and composing music [60, 61]. In this study, we have used “music medicine,” which is mistakenly called “music therapy” in Iranian studies.

Music medicine is effective through two mechanisms: capturing the patient's attention, engaging the mind with music, and distracting from pain. Exposure to engaging music can also affect mood and reduce stress levels [34, 35]. The second mechanism, which is more fundamental, involves the impact of music on the release of brain morphine, cortisol, and stress hormones [36].

Regarding acupressure, numerous studies affirm the effectiveness of this nonpharmacological method in reducing pain intensity. Ozkan et al. confirmed the efficacy of acupressure in reducing venipuncture pain in children [62]. Furthermore, Yildirim et al. conducted a study on “The Effect of Acupressure on Vital Signs, Acute Pain, and Stress during Venipuncture” involving 200 patients in Istanbul. They concluded that acupressure led to decreased pain and stress, with the experimental group patients reporting higher satisfaction scores than the control group [63]. Torkiyan et al. investigated the effect of acupressure on the GB21 point for labor pain in primiparous women and reported lower pain intensity in the experimental group [64]. In a study by Serce et al., acupressure was also found to reduce bone pain in cancer patients [65]. In a study by Pour et al. on children aged 6–12 years, the mean pain score was higher in the control group than in the acupressure and EMLA cream groups. However, there was no significant difference between the two experimental groups [66]. Variability in study outcomes can be attributed to differences in massage techniques, age groups studied, and variations in specific massage points targeted across different research investigations.

Based on the Gate Control Theory of Pain, stimulating the skin through massage can activate the large nerve fibers that transmit signals to the spinal cord, effectively keeping the pain gates closed and reducing pain sensation [67]. According to Chinese theory, the vital body energy known as “qi” flows through meridian channels regulating bodily functions. Blockages in these energy channels lead to disorders and pain. These channels can be accessed by massaging specific points on the body, resulting in energy balance and pain relief [68].

Some other studies have also reported the effectiveness of both music therapy and acupressure in reducing pain and anxiety. However, they found no significant difference between these methods [42, 69, 70]. In some cases, medicinal and nonmedicinal methods were more effective than music or acupressure, while music and acupressure yielded similar effects in others. Based on our research, no study has compared the effects of Hugo point massage and music therapy on the pain intensity of IV catheter insertion in young children. This lack of comparison arises from limited clinical trials in this field, challenging effective result comparisons.

### 4.1. Study Limitations

Some of the most critical limitations of the present study are as follows. First, this research was conducted on children aged 3 to 6 years; thus, its generalizability to all age groups is limited. Second, the study did not account for variables such as children's mood and socio-cultural background, which could potentially influence a child's perception of pain. These factors were beyond the researcher's control and represent a research limitation. Third, the child's stress levels may have affected their ability to engage with the distraction factor, but the study did not assess the impact of stress on pain.

Additionally, the study's internal validity was compromised due to the inability to implement blinding procedures. Because pain is a subjective phenomenon that relies on self-reported data, individuals may have varying perceptions of pain intensity, leading to potential overestimation or underestimation. Furthermore, the convenience purposive sampling method has limitations, including limited representativeness (external validity), potential for selection bias, lack of diversity, limited control over sample characteristics, and difficulty establishing causality. Indeed, researchers have attempted to reduce this method's drawbacks by randomly allocating individuals into three groups with the help of sealed envelopes. Therefore, it is advisable to conduct further research while considering the limitations identified in this study.

## 5. Conclusion

Based on the findings, it is evident that music therapy is a superior method in alleviating the discomfort associated with venipuncture compared to acupressure. Considering the paramount significance of effective pain management, recognized as the fifth vital sign, and the adverse consequences stemming from inadequate control thereof, the recommendation is to employ music therapy as the primary approach. Subsequently, Hugo advocates for incorporating acupressure techniques to mitigate the intensity of venipuncture-induced pain in pediatric patients.

While it is imperative to acknowledge that music therapy cannot replace pharmaceutical interventions directly, it is a valuable adjunctive and complementary therapeutic modality. Music therapy can potentially curtail the dependence on pharmacological remedies, including sedative agents, thereby underscoring its role in optimizing patient care.

## Figures and Tables

**Table 1:** The demographic characteristics of the participants in the music, acupressure, and control groups.

Groups	Music (*n* = 60)	Acupressure (*n* = 60)	Control (*n* = 60)	*p* value
Characteristics	*N* (%)	*N* (%)	*N* (%)	
Gender				
Male	29 (48.3)	29 (48.3)	30 (50)	*X* ^2*∗*^ = 0.044, 2, *p*=0.987
Female	31 (51.7)	31 (51.7)	30 (50)	
Age (years)				
<4	28 (46.7)	17 (28.3)	18 (30)	*H* ^*∗∗*^ = 3.08, 2, *p*=0.214
4–4.9	8 (13.3)	13 (21.7)	18 (30)	
≥5	24 (40)	30 (50)	24 (40)	
Underlying disease				
Positive	9 (15)	11 (18.3)	17 (28.3)	*X* ^2*∗*^ = 3.54, 2, *p*=0.170
Negative	51 (85)	49 (81.7)	43 (71.7)	
Causes of hospitalization				
Fever and seizure	20 (33.3)	12 (20)	12 (20)	*X* ^2*∗*^ = 5.48, 6, *p*=0.484
Gastroenteritis	16 (26.7)	20 (33.3)	23 (38.3)	
Respiratory disease	15 (25)	14 (23.3)	15 (25)	
Others	9 (15)	14 (23.3)	10 (16.7)	
History of the previous injection				
Positive	20 (33.3)	27 (45)	36 (60)	*X* ^2*∗*^ = 8.63, 2, *p*=0.013
Negative	40 (66.7)	33 (55)	24 (40)	

^*∗*^Chi-square test; ^*∗∗*^Kruskal–Wallis test.

**Table 2:** Distribution of absolute, relative frequencies and mean scores of the research units according to the intensity of pain during venipuncture in the music, acupressure, and control groups.

Groups	Music (*n* = 60)	Acupressure (*n* = 60)	Control (*n* = 60)	*p* value
Pain intensity	*N* (%)	*N* (%)	*N* (%)	
Mild (1–2.9)	20 (33.3)	3 (5)	0 (0)	

Moderate (3–6.9)	36 (60)	47 (78.3)	3 (5)	

Severe (7–9.9)	4 (6.7)	10 (16.7)	48 (80)	

Very severe (10)	0 (0)	0 (0)	9 (15)	

Mean pain score (mean ± SD)	3.32 ± 1.44	4.82 ± 1.51	8.32 ± 1.10	^*∗*^ *H* = 125.28, 2, *p* < 0.001
95% CI^*∗∗*^	2.94–3.69	4.43–5.21	8.03–8.60	

^*∗*^Kruskal–Wallis test; ^*∗∗*^confidence interval.

**Table 3:** Mean ± SD severity of pain during venipuncture in the music, acupressure, and control groups with and without a history of previous injection.

Groups	Music	Acupressure	Control	*p* value^*∗*^
Pain intensity	Mean ± SD	Mean ± SD	Mean ± SD	
With a history of previous injection	3.50 ± 1.28	5.04 ± 1.63	8.28 ± 1.21	*H* = 59.97, 2, *p* < 0.001
Without a history of previous injection	3.23 ± 1.53	4.64 ± 1.41	8.38 ± 0.92	*H* = 61.60, 2, *p* < 0.001

^*∗*^Kruskal–Wallis test; SD: standard deviation.

## Data Availability

The datasets used and/or analyzed during the current study are available from the corresponding author on reasonable request.
